# Epidemiological Characterization of Drug Resistance among *Mycobacterium tuberculosis* Isolated from Patients in Northeast of Iran during 2012-2013

**DOI:** 10.1155/2015/747085

**Published:** 2015-05-03

**Authors:** Ashraf Tavanaee Sani, Abolfazl Shakiba, Maryam Salehi, Hamid Reza Bahrami Taghanaki, Seiedeh Fatemeh Ayati Fard, Kiarash Ghazvini

**Affiliations:** ^1^Department of Infectious Diseases, Mashhad University of Medical Sciences, Mashhad, Iran; ^2^Departments of Community Medicine, School of Medicine and Research Center for Patient Safety, Mashhad University of Medical Sciences, Mashhad, Iran; ^3^Department of Traditional and Alternative Medicine, Mashhad University of Medical Sciences, Mashhad, Iran; ^4^Tabriz University of Medical Sciences, University Street, P.O. Box 5165665931, Tabriz, Iran; ^5^Antimicrobial Resistance Research Center, Buali Research Institute and Department of Microbiology and Virology, Faculty of Medicine, Mashhad University of Medical Sciences, Mashhad, Iran

## Abstract

*Introduction*. Tuberculosis is still one of the most important health problems in developing countries and increasing drug resistance is the main concern for its treatment. This study was designed to characterize the drug resistant *Mycobacterium tuberculosis* isolated from patients suffering from pulmonary tuberculosis in northeast of Iran. *Method*. In this cross-sectional study during 2012-2013, drug susceptibility testing was performed on *Mycobacterium tuberculosis* isolated in northeast of Iran using proportional method. Epidemiological data concerning these strains were also analyzed. *Results*. Among 125 studied isolates, 25 mycobacteria (20%) were diagnosed as nontuberculosis mycobacteria. Among the remaining 100 *Mycobacterium tuberculosis* isolates, the resistance rates were 7%, 7%, 3%, and 9% against isoniazid, rifampin, ethambutol, and streptomycin, respectively. Four isolates were resistant against both isoniazid and rifampin (MDR tuberculosis). The highest resistance rate was observed among 15–45-year-old patients. The MDR tuberculosis was much more prevalent among those who had previous history of treatment. *Conclusion*. Considering these findings, DOTS strategy should be emphasized and promptly used in order to prevent further resistance. Regarding the high rate of nontuberculosis mycobacteria, it is recommended that confirmatory tests were performed before any therapeutic decision.

## 1. Introduction

One-third of the world population is contaminated by* Mycobacterium tuberculosis*. Every year, about 9 million people suffer from active tuberculosis, and about 2 million die because of this disease [1]. In 2009, about 9.4 million new cases of tuberculosis were reported around the world. In the same year in Iran, 10,099 cases of tuberculosis were reported; among those 5,100 cases had positive sputum which were highly contagious [1]. Tuberculosis has been reported in all the provinces in Iran, but this rate was higher in eastern part, as neighboring to Afghanistan and Pakistan which have the highest rate of tuberculosis in the world.

Drug resistant tuberculosis is the main problem in controlling tuberculosis. In contrast to drug susceptible cases, patients who have been infected with drug resistant strains need two years of treatment instead of 6 months and the treatments are 70 times more costly in addition to much higher mortality rate (about 40–60%) [2]. According to World Health Organization, there were 9.2 million new cases of tuberculosis and 500 thousand cases of drug resistance in 2006. Also, World Health Organization and other international studies stated that the resistance against antituberculosis antibiotics exists all around the world, and the global prevalence of primary resistance is almost 10.7% [1, 3].* Mycobacterium tuberculosis* strains which are resistant against several drugs (MDR-TB) are also prevalent and cause high mortality rate in spite of treatment [4, 5]. In the past decade, MDR-TB has become the main threat for tuberculosis control strategies [6]. More attention was given to this issue when tuberculosis with extensive drug resistance (XDR) has been reported in some locations [7]. The latest reports indicate that, in Iran, there is a high rate of MDR and XDR and even resistance against all drugs (TDR) [8]. This study was designed to characterize the epidemiology of drug resistance among* Mycobacterium tuberculosis* isolates in northeast of Iran during 2012-2013 to plan better controlling strategies.

## 2. Method

In this study, all culture positive samples from patients with pulmonary tuberculosis in northeast of Iran during 2012 and 2013 were referred to Regional Reference Laboratory of Tuberculosis, Mashhad, Iran. All isolates were examined by Ziehl Neelsen staining and biochemical and phenotypic methods. Biochemical and phenotypic methods for identification of mycobacteria include observation of rate of growth, colony morphology, pigmentation, and biochemical profiles. Biochemical testing (i.e., niacin, nitrate reduction, and 68°C labile catalase tests) was used to definitively identify the isolated mycobacteria once they are categorized into a preliminary subgroup based on their growth characteristics [9, 10]. Also a multiplex polymerase chain reaction was carried out for confirmation as prescribed previously [11–14]. For this PCR assay, two different pairs of oligonucleotide primers targeting the gene encoding for 85 KDa protein (common to all mycobacteria) and the insertion sequence IS6110 (specific for* M. tuberculosis* complex) were used [11–14].

The susceptibilities of the isolates which were identified as* Mycobacterium tuberculosis* were determined against isoniazid, rifampicin, ethambutol, and streptomycin by standard proportional method using Lowenstein-Jensen medium as recommended by World Health Organization (WHO) [15]. For this purpose, several dilutions of inoculums (10^−2^ and 10^−4^) were planted onto both control and drug containing media. The proportion of resistant bacilli against given drug is then determined by comparing these numbers and expressing the resistant portion as a percentage. Susceptibility was defined as no or less than 1% growth on media containing the critical concentration of drug, and the resistance was defined as growth of 1% or more of the bacterial population. To ensure that results of drug susceptibility testing are reliable and accurate, the standard strain H37Rv and two strains of drug resistant* M. tuberculosis* were included in each series of testing [15]. Epidemiological data about the patients were gathered. The data was then reviewed and analyzed using SPSS.

## 3. Results

**Figure 1:**
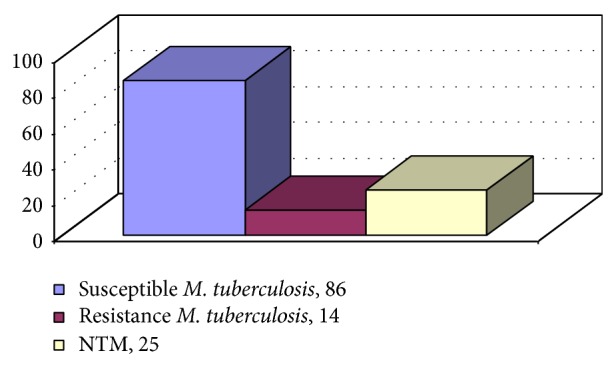
The distribution of mycobacteria studied in this research project.

Among 125 patients in this study, 25 samples were diagnosed as nontuberculosis mycobacteria by biochemical and molecular methods (Figure 1).

From 100 patients whose culture resulted in* Mycobacterium tuberculosis*, 86 isolates were susceptible to antituberculosis antibiotics, and 14 isolates were resistant. Among resistant isolates, 7 isolates (7%) were resistant against isoniazid, 7 isolates (7%) were resistant against rifampin, and 3 and 9 isolates were resistant against ethambutol and streptomycin, respectively. Four isolates were resistant against both isoniazid and rifampin. Three isolates (3%) were resistant against isoniazid and streptomycin and 4 isolates (4%) were resistant against rifampin and streptomycin. In this study, only two isolates (2%) were resistant against all 4 antibiotics (Table 1).

As it has been shown in Table 2, no* Mycobacterium tuberculosis *was isolated from people younger than 15 years old, and the most resistance cases were among 15–45-year-old groups.

As the epidemiological data of 18 patients were unclear, we performed all the analysis on the remaining 82 patients. Among these 82 patients who have been infected with* Mycobacterium tuberculosis*, there were 45 male patients and 37 female patients. The age range was between 16 and 94 years old, with the average 53 years old. Seventy-three patients were Iranian, and 9 patients were non-Iranian. Seventy-one patients live in urban areas and 11 in rural area. There were 56 new cases and 26 cases with previous history of treatment. The relations between demographic and epidemiological data with resistance and multiple drug resistance were analyzed and there was not any significant relation between these parameters and resistance (Table 3).

Interestingly, out of these 4 multiple drug resistant cases, 3 patients had a history of previous tuberculosis and treatment, so the rate of MDR tuberculosis among patients with relapse history was 11.53%. And only one patient diagnosed with MDR tuberculosis was a new case (1.78%) (Table 4).

Although the grading scale of smear positivity was highly associated with multiple drug resistant, the relation was not statistically significant (Table 5).

## 4. Discussion

Nowadays, drug resistance is the main problem in controlling tuberculosis in the world. During the last years, the resistance rate has been steadily increased. As the effective drugs are very limited, resistance is the main threat for STOP-TB programs. Traditionally, patients with drug resistant tuberculosis have been assumed that acquired drug resistance due to their previous treatment history. The term “acquired drug resistance” in patients with tuberculosis implies that resistance has developed during treatment [16].

In this study, the resistance rates of* Mycobacterium tuberculosis* against isoniazid, rifampin, ethambutol, and streptomycin were 7%, 7%, 3%, and 9%, respectively. The rate of MDR tuberculosis was 4% which is similar to the rate obtained in another study in Mashhad during 2008 in which the rate of MDR tuberculosis was 4.65%. Our resistance rate seems very close to the rate reported by Dr. Velayati and colleagues. They reported 5.6% MDR tuberculosis among 146 patients suffering from tuberculosis in Iranian National Reference TB Laboratory [17]. As they received sample from all over the country, this rate sounds highly reliable.

However, some researchers reported lower rate for resistance in Iran. In Shamaei's study on 546 patients in Tehran, 2.8% people were reported with MDR [18]. And also in Hadizadeh and colleagues' study in Tehran between 2006 and 2009, the resistance against isoniazid and rifampin was 11% and 10%, respectively, and 2.5% of cases were MDR tuberculosis [19]. This lower rate of resistance might be due to obtaining sample from restricted regions with low rate of resistance. On the contrary, the reported rate for resistance from southeast of Iran is much higher than our results. For example, Metanat and colleagues reported 27%, 33%, 39%, and 55% resistance rate against rifampin, isoniazid, ethambutol, and streptomycin, respectively, as they conducted a study on 84* Mycobacterium tuberculosis* isolates in Buali Hospital in Zahedan (southeast of Iran). The rate of MDR was 16% in their study [20]. In another study, during 2012 by Metanat and colleagues, the multidrug resistance rate of 12% was reported for tuberculosis in Zahedan (southeast of Iran) among 88 tuberculosis cases [21]. This high rate of resistance can be interpreted by considering neighboring of that region with Afghanistan and Pakistan as the most prevalent area for tuberculosis.

In one study in Uzbekistan and Kazakhstan, drug resistance was reported as 13%, and in China, it was reported as 10% [22]. A study in Pakistan during 2009–2011 showed that the resistance rate against isoniazid was 15.5% and simultaneous resistance against isoniazid, pyrazinamide, and ethambutol was 1% [23]. In another study, in Saudi Arabia during 2012, the resistance rate against isoniazid was 33%, and the resistance against rifampin, streptomycin, and ethambutol was 23%, 13%, and 3%, respectively. In this study, the resistance rate against two drugs was 20% [24]. Considering these data, our resistance rate is much lower which could be due to good tuberculosis controlling measures. For example, tuberculosis treatment is free of charge in Iran and paid completely by government. Our results confirmed that prompt implementation of DOTS strategy in our region has slowed down the increasing incidence of resistant tuberculosis and has prevented a widespread resistance in this community.

Around the world, the prevalence of MDR among pulmonary tuberculoses is 1.4% in new cases and 13% in recurrence of tuberculosis [25]. Our findings show that in northeast of Iran the prevalence of MDR in new cases of pulmonary tuberculosis was 1.78 and among patients with history of relapse was 11.53%. Our rates are very similar to the global rate of resistance in new and recurrent cases.

Another notable finding of this study is the high prevalence of nontuberculosis mycobacterium (NTM) consisting of 20% of the cases. Such patients often were treated as tuberculosis which did not respond and categorized as treatment failure. This means that, in addition to receiving inappropriate treatment, improper antituberculosis antibiotics were also used. This could cause an increased resistance against antituberculosis antibiotics, especially isoniazid and rifampin. Also in our study, the resistance against streptomycin was much higher than other drugs which is expected because of using this drug for other diseases.

## 5. Conclusions

As northeast of Iran is in neighborhood of Afghanistan and Turkmenistan which are countries with a very high burden of disease and also high rate of resistant tuberculosis, prompt preventive policies and complete DOTS strategy should be implemented in order to prevent more incidences of resistance.

Given the high rate of nontuberculosis mycobacterium, it is recommended that treatment starts after accurate diagnostic procedure in order to prevent improper drug usage and also to prescribe proper treatment from the beginning.

## Figures and Tables

**Table 1:** Pattern of drug resistance among *Mycobacterium tuberculosis* isolates in northeast of Iran during 2012-2013.

	Pattern of resistance against each antibiotic for those 14 resistant isolates													
	Resistant isolate 1	Resistant isolate 2	Resistant isolate 3	Resistant isolate 4	Resistant isolate 5	Resistant isolate 6	Resistant isolate 7	Resistant isolate 8	Resistant isolate 9	Resistant isolate 10	Resistant isolate 11	Resistant isolate 12	Resistant isolate 13	Resistant isolate 14
Isoniazid	∗	∗	∗	∗	∗				∗	∗				
Rifampin	∗	∗	∗	∗		∗	∗	∗						
Ethambutol	∗	∗	∗											
Streptomycin	∗	∗			∗	∗	∗				∗	∗	∗	∗
History of previous treatment for TB	∗	∗		∗	∗	∗	∗	∗		∗	∗			

**Table 2:** The distribution of drug resistance by age.

Age range	Number	Sensitive cases	Resistant cases	Percentage
<15	0	0	0	0
45–15	36	28	8	58%
65–45	15	13	2	14%
>65	31	27	4	28%

Total	82	68	14	100%

**Table 3:** The demographic data of patients.

Demographics status	Condition	Total	Sensitive	Resistant	MDR-TB	*P* value	NTM
Sex	Male	45	37	8	2	0.749	5
	Female	37	31	6	2		13

Nationality	Iranian	73	61	12	3	0.301	18
	Non-Iranian	9	7	2	1		0

Living area	Urban	71	59	12	3	1	15
	Rural	11	9	2	1		3

History of previous treatment	Yes	26	19	7	3	0.066	6
	No	56	49	7	1		12

Diabetes	Yes	11	11	0	0	0.344	5
	No	71	57	14	4		13

Smoking	Yes	9	7	2	0	0.301	1
	No	73	61	12	4		17

Family history of TB	Yes	15	12	3	1	1	3
	No	67	56	11	3		15

Opium addiction	Yes	12	8	4	0	1	0
	No	70	60	10	4		18

History of imprisoning (jail)	Yes	25	22	3	1	0.635	0
	NO	57	46	11	3		18

NTM: nontuberculosis mycobacterium, MDR TB: multiple drug resistant tuberculosis.

**Table 4:** Percentage of MDR tuberculosis in new cases and patients with relapse during 2012-2013.

	Total number of cases	MDR cases
History of previous treatment	26	3 (11.53%)
New case	56	1 (1.78%)

**Table 5:** Grading scale of smear positivity in sensitive and resistant isolates.

Grading scale of smear positivity	Sensitive	Resistance	*P* value
1+	22 (32%)	4 (28%)	0.056
2+	11 (16%)	1 (7%)	
3+	35 (52%)	9 (65%)	

Total	68 (100%)	14 (100%)	
